# Eyes ahead: a scoping review of technologies enabling humanoid robots to follow human gaze

**DOI:** 10.3389/frobt.2025.1723527

**Published:** 2026-01-16

**Authors:** Leana Neuber, Wolf Culemann, Ruth Maria Ingendoh, Angela Heine

**Affiliations:** University of Duisburg-Essen, Department of Psychology, Essen, Germany

**Keywords:** gaze following, human-robot interaction, joint attention, scoping review, shared gaze, social robot, triadic interaction

## Abstract

Gaze is a fundamental aspect of non-verbal communication in human interaction, playing an important role in conveying attention, intentions, and emotions. A key concept in gaze-based human interaction is joint attention, the focus of two individuals on an object in a shared environment. In the context of human–robot interaction (HRI), gaze-following has become a growing research area, as it enables robots to appear more socially intelligent, engaging, and likable. While various technical approaches have been developed to achieve this capability, a comprehensive overview of existing implementations has been lacking. This scoping review addresses this gap by systematically categorizing existing solutions, offering a structured perspective on how gaze-following behavior is technically realized in the field of HRI. A systematic search was conducted across four databases, leading to the identification of 28 studies. To structure the findings, a taxonomy was developed that categorizes technological approaches along three key functional dimensions: (1) environment tracking, which involves recognizing the objects in the robot’s surroundings; (2) gaze tracking, which refers to detecting and interpreting human gaze direction; and (3) gaze–environment mapping, which connects gaze information with objects in the shared environment to enable appropriate robotic responses. Across studies, a distinction emerges between constrained and unconstrained solutions. While constrained approaches, such as predefined object positions, provide high accuracy, they are often limited to controlled settings. In contrast, unconstrained methods offer greater flexibility but pose significant technical challenges. The complexity of the implementations also varies significantly, from simple rule-based approaches to advanced, adaptive systems that integrate multiple data sources. These findings highlight ongoing challenges in achieving robust and real-time gaze-following in robots, particularly in dynamic, real-world environments. Future research should focus on refining unconstrained tracking methods and leveraging advances in machine learning and computer vision to make human–robot interactions more natural and socially intuitive.

## 1 Introduction

Understanding the dynamics of human interaction goes beyond what is said, with gaze serving as an important non-verbal channel through which people interact (Kim et al., 2008; Saran et al., 2018; Matsumoto and Zelinsky, 1999). Central to understanding gaze-based human interaction is the concept of *social gaze* which refers to eye movements conveying factual, intentional or affective content, either intentionally or non-intentionally (Admoni and Scassellati, 2017). In dyadic interactions, the social exchange between two individuals, mutual gaze is seen as a core component of the interaction process. In triadic interactions, which involves two individuals and a third object or subject, the dynamics of social gaze becomes more complex. Jording et al. (2018) suggested five possible interactional states that a person can adopt in triadic interactions:

1. Partner-oriented: The person focuses on the other person.
2. Object-oriented: The person focuses an object in the shared environment.
3. Introspective: The person focuses on inner thoughts and processes.
4. Responding joint attention: The person follows the gaze of the other person to an object in the shared environment.
5. Initiating joint attention: The person tries to get the other person to direct their gaze and thus their attention toward an object in the shared environment.

One aspect of human–human interaction (HHI) is, thus, joint attention which refers to the ability of humans to follow someone else’s gaze and share the other person’s focus on an object–a human skill which is learned in early infancy (Emery, 2000). Common orientation on an object or a person in the environment has been studied not only in HHI but also in human–robot interaction (HRI) as a possible mechanism to enhance the naturalness and effectiveness of interactions between humans and robots. However, given that joint attention presupposes the capacity to interpret the goals, intentions, and beliefs of others based on their behavior (Scassellati, 2002), and given that robots do not (yet) possess these human social cognitive capacities, it is more accurate to refer to the respective behavioral patterns of robots as gaze following behavior. So, gaze following in human–robot triadic interactions means that the robot is programmed to recognize the human’s gaze target and react in an appropriate way. To this effect, Grigorescu and Macesanu (2017) refer to what they call a social bridge between human and artificial interaction partners that can be established when social robots are enabled to follow the gaze of humans in real time. Meanwhile, there are a number of studies that show that robots which mimic human gaze behavior are perceived as more socially present, engaging, and likable (Xu et al., 2013; Willemse and Wykowska, 2019; Pereira et al., 2019).

With regard to the technological prerequisites for gaze following, the robot must first be enabled to track and interpret its human partner’s gaze. To date, the most precise solution to gauge human gaze trajectories involves the use of eye tracking devices which calculate gaze direction with high accuracy from the corneal reflections of emitted infrared light. An alternative solution for gaze tracking is based on the use of computer vision algorithms which detect head position and gaze direction without additional hardware apart from the robot’s built-in or external cameras. In fact, recent advances in computer vision and machine learning have led to considerable improvements in camera-based gaze direction detection (Su et al., 2023; Cazzato et al., 2020). Apart from tracking the gaze of the human interaction partner, the robot must also be able to match the human’s gaze vector with objects or persons in a shared environment in order to be able to react in a contingent way (Zhang et al., 2020). Both aspects of gaze following behavior constitute major technological challenges in the context of real-time applications.

In order to consolidate previous research in this domain, Admoni and Scassellati (2017) published a comprehensive review of the available approaches to gaze following in HRI. The authors summarize studies that focus on user experience and behavior of participants who interact with robots capable of following human gaze. Additionally, and of relevance for the present study, the authors provide a general overview of the technological processing steps required to enable robotic gaze following in HRI. However, while they outline the key concepts underlying robotic gaze following, they do not discuss technical implementation approaches in depth. So, to the best of our knowledge, no overview of technological advancements in equipping robots with gaze following capacities is currently available.

### 1.1 Objective

The present scoping review aims at closing this gap. To this end, the scientific literature was scanned, and relevant technological solutions for gaze following in social robots were identified. In order to allow for a comprehensive overview of the state of the art in this field of research, a taxonomy was developed that systematizes current technological approaches to the core components of robotic gaze following, namely, environment tracking, gaze tracking, and gaze–environment mapping. This allows for the identification of trends as well as open problems related to gaze following capacities in robots and provides the basis for recommendations for future research and development.

## 2 Methods

This scoping review follows the general approach suggested by the PRISMA 2020 statement (Preferred Reporting Items for Systematic Reviews and Meta-Analyses; Page et al. (2021)), as well as the PRISMA extension for scoping reviews (Tricco et al., 2018). The following sections describe the database search, inclusion and exclusion criteria, and the final literature selection in detail.

### 2.1 Search

An initial systematic literature search was conducted in January 2025. A second search in August 2025 was conducted to update the data. Both searches were performed using the databases Scopus^1^, IEEE^2^, Web of Science (WoS)^3^, and PubMed^4^. The searches were restricted to publications in English and German without limitations regarding the publication date. A broad search was conducted to present the current state of the development. To this end, the search terms included the terms eye, gaze or head movement within the context of human–robot interaction, collaboration or cooperation. Additionally, to cover gaze-contingent robot behavior the search included the terms shared, synchronized, responding, following and imitating behaviors in HRI. Table 1 provides a detailed summary of the databases and the search queries, as well as the number of retrieved records. The results were screened using a reference management database (Zotero^5^, Corporation for Digital Scholarship, Vienna, Virginia).

**TABLE 1:** Details of the search process for each database as of 24.08.2025.

Database	Syntax	Number retrieved
Scopus	TITLE-ABS-KEY ((“gaze” OR “eye” OR “head”) AND (“human* robot” OR “child robot” OR “cri” OR “hri” OR “social robot*“) AND (“interact*” OR “collaborat*” OR “cooperat*“) AND (“joint” OR “engage*” OR “coordinat*” OR “share*” OR “sync*” OR “respon*” OR “follow” OR “pursu*” OR “mimick*” OR “imitat*“))	1,399
IEEE	((“All Metadata”:“gaze” OR “all Metadata”:“eye” OR “all Metadata”:“head”) AND (“all Metadata”:“human* robot” OR “all Metadata”:“child robot” OR “all Metadata”:“CRI” OR “all Metadata”:“HRI” OR “all Metadata”:“social robot”) AND (“all Metadata”:“interact*” OR “all Metadata”:“collaborat*” OR “all Metadata”:“cooperat*“) AND (“all Metadata”:“joint” OR “all Metadata”:“engage” OR “all Metadata”:“coordinat*” OR “all Metadata”:“share*” OR “all Metadata”:“sync*” OR “all Metadata”:“respon*” OR “all Metadata”:“follow” OR “all Metadata”:“pursu*” OR “all Metadata”:“mimic” OR “all Metadata”:“imitat”))	1,373
Web of science	TS=((“gaze” OR “eye” OR “head”) AND (“human* robot” OR “child robot” OR “cri” OR “hri” OR “social robot”) AND (“interact*” OR “collaborat*” OR “cooperat*“) AND (“joint” OR “engage*” OR “coordinat*” OR “share*” OR “sync*” OR “respon*” OR “follow” OR “pursu*” OR “mimick*” OR “imitat*“))	436
PubMed	(“gaze” [Title/Abstract] OR “eye” [Title/Abstract] OR “head” [Title/Abstract]) AND (“human* robot” [Title/Abstract] OR “child robot” [Title/Abstract] OR “cri” [Title/Abstract] OR “hri” [Title/Abstract] OR “social robot*“ [Title/Abstract]) AND (“interact*“ [Title/Abstract] OR “collaborat*“ [Title/Abstract] OR “cooperat*“ [Title/Abstract]) AND (“joint” [Title/Abstract] OR “engage*“ [Title/Abstract] OR “coordinat*“ [Title/Abstract] OR “share*“ [Title/Abstract] OR “sync*“ [Title/Abstract] OR “respon*“ [Title/Abstract] OR “follow” [Title/Abstract] OR “pursu*“ [Title/Abstract] OR “mimick*“ [Title/Abstract] OR “imitat*“ [Title/Abstract])	144

### 2.2 Inclusion and exclusion criteria

Several inclusion and exclusion criteria were applied. For inclusion into the current review, studies were required to: (a) involve neurologically healthy adults or children; (b) use camera-based approaches, eye tracking devices or motion capture systems for tracking, monitoring or estimating human gaze, head posture, orientation or position; (c) include an implemented solution where the robot not only detects the user’s orientation, but also tracks or recognizes objects in the shared environment; (d) present a solution implemented on a humanoid robot; (e) demonstrate that the robot is capable of reacting, responding, adapting or changing its behavior in real time or has the potential to do so, in contingency with the tracked posture, gaze, orientation or position and the identified object(s); (f) be conducted in social-interactive contexts; and (g) be original, published research using an original dataset. For the purpose of this review, a *humanoid robot* is defined as a physically embodied robotic system whose morphology is close to the human body, featuring a human-like head–torso structure and typically one or more human-like limbs. Under this definition, robotic heads qualify when they form part of a human-like body schema, whereas non-anthropomorphic settings, like standalone cameras on mounts, or toy-like social robots are excluded. Additionally, studies were excluded if: (a) participants with neurological disorders were involved; (b) no embodied robot was used, including studies in virtual environments; (c) the study was conducted in a medical context, e.g., studies on surgical robots; (d) the context of the study was industrial, e.g., studies on robotic arms; (e) a Wizard of Oz study setting was employed, where robots are controlled remotely by humans; or (f) the publication was not in English or German.

### 2.3 Literature selection

Initially, the search identified 3,352 records. Three additional studies were found by a secondary citation search, resulting in the total number of 3,355 publications. After removing duplicates and entries with insufficient data, 2,333 titles and abstracts were screened, and the exclusion and inclusion criteria applied. Screening and study selection were conducted by the first author (L.N.). An independent rater (R.M.I.) repeated the search and screening. All differences in retrieved studies were resolved through discussion. Data extraction was performed by the first author (L.N.).

**FIGURE 1:**
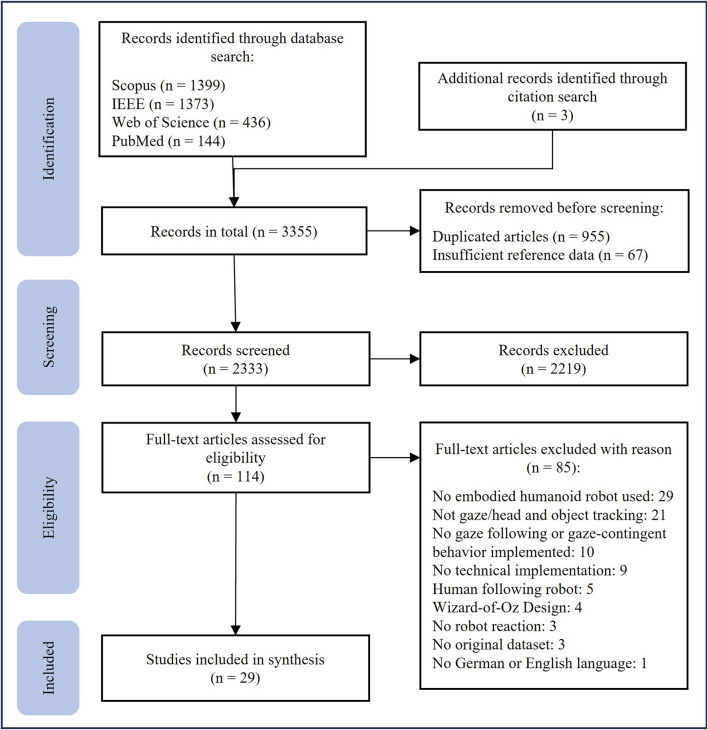
PRISMA-ScR flow diagram for the search process (Tricco et al., 2018).

From the initial set, 2,219 records were excluded during the title and abstract screening, narrowing the results down to 114 studies. The large number of exclusions at this stage can be attributed to the application of the exclusion criteria. Because the decisions at this phase were based on title and abstract only, multiple exclusion reasons could apply to a single record. To improve transparency, we nevertheless assigned exclusion reasons to broad categories whenever possible. For instance, a significant number of studies involve participants diagnosed with neurological disorders (n = 144), which did not meet the exclusion criterion (a). An overview of the studies in this area, however, is provided by Chevalier et al. (2020). Additionally, a large number of studies examine robots operating in industrial or medical contexts (e.g., robotic arms or surgical systems; n = 295), where the primary goal is task execution rather than gaze following in social HRI. Furthermore, the search query does not distinguish between studies where the robot induces gaze following behavior, i.e., the human interaction partner is following the robot’s gaze, and studies in which the robot follows the human’s gaze. Such studies (n = 34) were also excluded as they did not align with the scope of this review. A detailed list of all screened records, including first- and second-stage (full-text) screening decisions and the corresponding exclusion reasons, is provided in the Appendix. Following the title and abstract screening phase, the inclusion and exclusion criteria were applied again to the remaining 114 studies based on their full texts, resulting in 29 studies synthesized in this review. Figure 1 presents a flow diagram of the entire process, structured in accordance with the PRISMA-ScR guidelines. The study was not pre-registered.

## 3 Results

**FIGURE 2:**
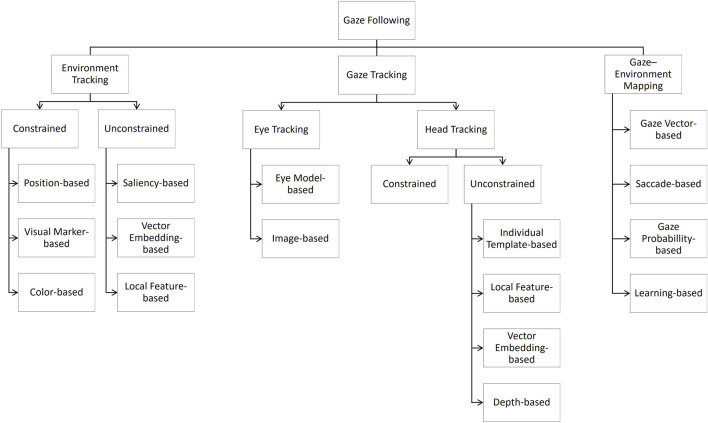
Taxonomy of technical contributions to gaze following systems.

To integrate the findings of the present systematic review, we suggest a taxonomy to systematize existing solutions with respect to their technical contributions to three basic functional dimensions of gaze following systems (*cf.* Figure 2). First of all, the robot must be able to process the environment, i.e., the general context, and relevant objects potentially addressed in triadic interaction have to be recognized. Secondly, the robot must recognize the human gaze. And, finally, the human gaze must be mapped to information about the environment. The latter, i.e., the gaze–environment mapping is the basis for a robot to be able to react in an appropriate way, for example, by referring to the objects in the environment the human partner focuses on.

This taxonomy serves as a framework for an analysis and conceptual integration of the studies included in the present review. Furthermore, by determining the contributions of the included studies with respect to the three functional dimensions of gaze following systems, potential research gaps and opportunities for future research can be identified. Table 2 gives an overview of how each study contributes to one or more of the three functional dimensions of gaze following systems. We note that some categories in our taxonomy capture the degree of constraint required for reliable operation (e.g., prepared vs. unprepared environments), which often reflects practical limitations or experimental necessities rather than a explicit design choices. Table 3 lists the relevant details of each empirical study, such as the type of robot used, the number of participants involved, the outcome measures assessed, and the key results with regard to the outcome measure. Where *N/A* is reported for robot, the type was not explicitly specified in the publication; however, available photographs or figures of the robot confirmed that the study met our inclusion criteria.

**TABLE 2:** Detailed categorization of gaze following studies.

Authors	Environment tracking	Gaze tracking	Gaze–environment mapping
Scassellati (2003)	Saliency-based	Individual template-based	Gaze vector-based
Nagai et al. (2003)	Saliency-based	Individual template-based	Learning-based
Hosada et al. (2004)	Saliency-based	Unconstrained head tracking	Learning-based
Nagai (2005)	Color-based	Individual template-based	Learning-based
Shon et al. (2005)	Saliency-based	Local-feature-based	Gaze vector-based
Yoshikawa et al. (2006a)	Position-based	Eye-model-based	N/A
Yoshikawa et al. (2006b)	Position-based	Eye-model-based	N/A
Kim et al. (2008)	Local-feature-based	Local-feature-based	Learning-based
Marin-Urias et al. (2009)	Visual marker-based	Visual marker-based	Gaze vector-based
Ravindra et al. (2009)	Position-based	Image-based	Gaze vector-based
Lohan et al. (2011)	Visual marker-based	Unconstrained head tracking	N/A
Da Silva and Romero (2012)	Saliency-based	Individual template-based	Learning-based
Xu et al. (2013)	Color-based	Eye-model-based	Gaze vector-based
Yücel et al. (2013)	Saliency-based	Local-feature-based	Gaze probability-based
Domhof et al. (2015)	Saliency-based	Eye-model-based	Gaze probability-based
Palinko et al. (2015)	Position-based	Image-based	Gaze vector-based
Palinko et al. (2016)	Position-based	Image-based and local-feature-based	Saccade-based
Fischer and Demiris (2016)	Vector-embedding-based	Depth-based	Gaze vector-based
Saran et al. (2018)	Vector-embedding-based	Local-feature-based	Gaze probability-based
Mahzoon et al. (2019)	Position-based	Visual marker-based	Learning-based
Pereira et al. (2019)	Visual marker-based	Unconstrained head tracking	N/A
Willemse and Wykowska (2019)	Position-based	Eye-model-based	Gaze vector-based
Pereira et al. (2020)	Visual marker-based	Unconstrained head tracking	N/A
Deng et al. (2021)	Saliency-based	Vector-embedding-based	Gaze vector-based
Lombardi et al. (2022)	Unconstrained	Unconstrained head tracking	Gaze vector-based
Jin et al. (2022)	Unconstrained	Unconstrained head tracking	Gaze probability-based
Raković et al. (2024)	Color-based	Eye-model-based	Gaze vector-based
Hanifi et al. (2024)	Vector-embedding-based	Vector-embedding-based	Gaze probability-based
Samaniego et al. (2024)	Vector-embedding-based	Vector-embedding-basedd	Gaze vector-based

**TABLE 3:** Study setup details of included studies.

Authors	Robot	Participants	Outcome measure	Result
Scassellati (2002)	Cog	1	N/A	N/A
Nagai et al. (2003)	N/A	1	Accuracy	61%–100%
Hosada et al. (2004)	N/A	1	Accuracy	80%
Nagai (2005)	Infanoid	1	Accuracy	90%
Shon et al. (2005)	Biclops	1	Accuracy	90%
Yoshikawa et al. (2006a)	Robovie-R2	33	Questionnaire	Stronger feeling of being looked at
Yoshikawa et al. (2006b)	Robovie-R1	39	Questionnaire	Stronger feeling of being looked at
Kim et al. (2008)	Robotic head	1	Accuracy	59%–78%
Marin-Urias et al. (2009)	HRP2	1	N/A	N/A
Ravindra et al. (2009)	Hope-3	10	Accuracy	80%
Lohan et al. (2011)	iCub	12	Video analysis	Stronger orientation toward the robot
Da Silva and Romero (2012)	WHA8030	1	Accuracy	72,89%
Xu et al. (2013)	NAO	21	Face looks, speech, attentional shifts	High sensitivity for robot gazing behavior
Yücel et al. (2013)	NAO	4	Location precision	0,18–0,52
Domhof et al. (2015)	LEA	10	Accuracy	67,8–96,7%
Palinko et al. (2015)	iCub	7	Accuracy	69,9%
Palinko et al. (2016)	iCub	10	Accuracy	60,6% (head tracking); 81,5% (eye tracking)
Fischer and Demiris (2016)	iCub	6	Accuracy/horizontal error	100%, 0,08–0,61 m
Saran et al. (2018)	N/A	10	F1-score	0.345–0.577
Mahzoon et al. (2019)	Infant robot	48	Accuracy	81%–100%
Pereira et al. (2019)	Robotic head	22	Questionnaire	Improved social presence
Willemse and Wykowska (2019)	iCub	37	Return-to-face saccades, questionnaires	Preference toward the robot
Pereira et al. (2020)	Robotic head	40 + 27	Questionnaire	Improved social presence for external observers, none for children
Deng et al. (2021)	Rehabilitation robot	5	AUC	0,92
Lombardi et al. (2022)	iCub	1	Mean average precision	68%–84.4%
Jin et al. (2022)	Service robot	N/A	AUC	0,88–0,9
Raković et al. (2024)	iCub	1	Accuracy	75%
Hanifi et al. (2024)	iCub	1	Accuracy	79,5%
Samaniego et al. (2024)	Pepper	1	F1-score	0.694

### 3.1 Environment tracking

The first functional dimension of gaze following systems focuses on the capacity to track the environment so as to enable the robot to recognize its surroundings and the objects within it. Approaches can be subdivided into whether the tracked environment is constrained and unconstrained.

#### 3.1.1 Constrained environments

Constrained environments are interaction contexts in which the objects are specifically prepared, arranged, or designed to facilitate gaze following. Consequently, the proposed solutions are not directly applicable to environment tracking in real-world contexts. Nevertheless, considering constrained environments is important, as they tend to be less susceptible to interference from confounding factors such as, e.g., changing lighting conditions. In addition, they serve as proof-of-concept approaches by providing straightforward solutions for laboratory settings. Three types of object tracking in constrained environments can be distinguished, i.e., position-based, visual marker-based, and color-based solutions.

Position-based. The simplest approach to constrained environment tracking is based on establishing fixed positions for relevant objects. For instance, by placing objects left, right and center, human gaze shifts in any of these general directions can be interpreted as an indication of the object being in the focus of attention. Seven of the included studies report this type of constrained environment, four of which place objects on the left and right of the table or in the robot’s hand (Yoshikawa et al., 2006b; Yoshikawa et al., 2006a; Palinko et al., 2015; Palinko et al., 2016). The other three solutions include the center as a third direction (Ravindra et al., 2009; Mahzoon et al., 2019; Willemse and Wykowska, 2019).

Visual Marker-based. Another method for tracking objects in the environment involves the use of visual markers. These visual markers, attached to the objects, can be captured by a camera and processed by computer vision algorithms so as to determine their location. Four of the included studies use this approach. Lohan et al. (2011) and Pereira et al. (2019), Pereira et al. (2020) use ARToolkit^6^ markers to track objects in the three-dimensional space. Marin-Urias et al. (2009) do not specify which type of marker they chose.

Color-based. Color-based computer-vision techniques identify and segment objects based on color and contrast features. To this end, the objects must have unique, uniform colors that stand out from the context. Nagai (2005), for instance, uses a yellow-colored object that contrasts the environment. Xu et al. (2013) use several objects with bright colors in a white room. The two robots used in this study are also assigned colors. Raković et al. (2024), trains a model for color recognition to allow for object detection.

#### 3.1.2 Unconstrained environments

Unconstrained environments mimic real-world conditions, where objects can hardly be tagged beforehand, positioned statically, or painted in specific colors. Consequently, unconstrained solutions are appropriate for robotic applications in settings such as schools or nursing homes, where adaptability to the natural environment is crucial. Here, approaches that recognize objects based on their saliency, vector representation, or local properties can be distinguished. The study by Jin et al. (2022) works in an unconstrained setting. However, it does not fit into one of the subcategories used here, since the method primarily predicts a gaze target location (heatmap) in the scene rather than describing how the attended object is recognized.

Saliency-based. One popular approach to object tracking in unconstrained environments is inspired by human visual processing and utilizes saliency differences to identify areas most likely to attract human attention. To this end, a saliency map is created, assigning a distinct value to each image pixel based on visual attributes such as color, motion, brightness, or orientation. By analyzing the resulting map, areas of interest or objects are identified based on their saliency values and position. Multidimensional saliency approaches are inspired by human visual processing and combine several image attributes to identify areas of salience. A frequently used solution for creating saliency maps was developed by Itti et al. (1998), who implemented a neural network to combine the dimensions of color, intensity, and orientation. In contrast, one-dimensional saliency-based approaches calculate saliency of the environment using a single dimension such as color. Saliency-based methods are used extensively across the studies we included in the review. Four studies implement the model developed by Itti et al. (1998), Shon et al. (2005) use the three dimensions originally proposed by Itti et al., while Domhof et al. (2015) introduce an additional depth dimension, and Scassellati (2002) a motion dimension. Yücel et al. (2013) evaluate the model of Itti et al. against two alternatives: the approach of Judd et al. (2009), which introduces further saliency dimensions, and the approach used by Bruce and Tsotsos (2009), which incorporates interdependency among individual pixels in an image. Hosada et al. (2004) and Nagai et al. (2003) do not specify which model they employ for saliency calculation but name the dimensions color, edges, and object movement. Deng et al. (2021) use the OpenCV^7^ software library to create a saliency map and add the depth dimension. The authors do not specify which underlying algorithm OpenCV applies for the calculation. Finally, Da Silva and Romero (2012) implement a one-dimensional solution that considers only color variations within the environment.

Vector Embedding-based. This category includes solutions that learn high-dimensional vector representations (embeddings) of the environment and objects within. The respective approaches involve the training of deep learning models, particularly Convolutional Neural Networks (CNN), to generate embeddings that encode features/characteristics of the environment such as color, texture, and spatial positioning (Albawi et al., 2017). After training, the networks are capable to create embeddings for new, previously unseen images. Objects are recognized and classified by comparing their unique embedding vectors through similarity metrics and probability analysis. Five studies included in this review employ CNNs for object recognition. Fischer and Demiris (2016) utilize a deep CNN alongside a Support Vector Machine (SVM) for classification. Hanifi et al. (2024) implement a solution based on a Mask R-CNN, following the architecture proposed by Ceola et al. (2021). Similarly, Lombardi et al. (2022) implement a Mask R-CNN-based object detection solution following the on-line object detection pipeline introduced by Maiettini et al. (2020). Finally, Saran et al. (2018) and Samaniego et al. (2024) apply the YOLO object detection algorithm, which is based on a CNN architecture (Redmon et al., 2015).

Local Feature-based. These solutions extract the local characteristics of objects within a context incrementally by, e.g., using methods like corner detection and texture analysis, and by subsequently clustering these features for object classification. Kim et al. (2008) use a local feature-based approach, which initially detects edges, and, in a second step, local features. A classification of the objects is achieved through k-means clustering.

### 3.2 Gaze tracking

The tracking of human gaze constitutes the second functional dimension of gaze-following systems. Publications in this dimension can be divided into distinct categories, too. While some rely on eye-tracking devices to directly measure gaze direction, the majority estimate it indirectly by using head orientation as a proxy for gaze direction.

#### 3.2.1 Eye tracking

Eye tracking approaches can be subdivided into physiological or eye model-based and image-based approaches, which differ in terms of the recording modality and the internal algorithms used for analysis. Furthermore, these approaches can also be classified as either constrained or unconstrained, depending on whether they require calibration or specialized setups.

Eye Model-based. Eye model-based approaches to gaze tracking rely on geometric models of the eye. Cameras record eye images and apply these models to calculate the position of the pupil in each single frame. Model-based techniques include the Pupil Center Corneal Reflection (PCCR) method, which uses infrared (IR) light to evoke light reflections on the cornea on the basis of which gaze direction is determined using an eye model (Xu et al., 2023). These methods generally fall into the constrained category, as they require user-specific calibration and often involve specialized equipment. Of the studies included here, six employ infrared (IR)-based eye trackers for gaze tracking. While Yoshikawa et al. (2006a), Yoshikawa et al. (2006b) use a remotely positioned NAC EMR-8B eye tracker (Nac Image Technology Inc.) with an unspecified sampling frequency, the other studies use head-mounted eye trackers: Eye-Trac 6,000 (ASL LLC) with a 30 Hz sampling frequency (Xu et al., 2013), Pupil Labs with a 30 Hz sampling frequency (Domhof et al., 2015) and a 120 Hz frequency (Raković et al., 2024), and Tobii Pro Glasses 2 with a 100 Hz sampling frequency (Willemse and Wykowska, 2019).

Image-based. Image or appearance-based methods frequently rely on off-the-shelf cameras and determine gaze direction by analyzing images of the face and eyes using computer vision or machine learning algorithms. These methods are typically unconstrained, as they do not require external light sources, specialized hardware, or individual calibration (Xu et al., 2023). Image-based solutions are used by three studies. Ravindra et al. (2009) use the FaceLab system^8^, but do not further specify the algorithm. Palinko et al. (2016) compare eye tracking with head tracking and, following Palinko et al. (2015), use the Dlib library^9^ to extract facial landmarks.

#### 3.2.2 Head tracking

The majority of the included studies approximate gaze direction by extracting the position of the head. In line with the classification for the approaches to environment tracking, the solutions are divided into either constrained or unconstrained, depending on the extent of preparation required.

##### 3.2.2.1 Constrained

Solutions that require prior preparation to calculate the head position are referred to as constrained. One example are motion capture systems that are based on optical markers attached to the participants’ heads. Cameras are used to track the markers and calculate the head position. This type of motion-capture systems is used by Marin-Urias et al. (2009) and Mahzoon et al. (2019).

##### 3.2.2.2 Unconstrained

In contrast, unconstrained solutions can be used in open environments without prior preparation of, e.g., the participants. These solutions include software-based methods that use standard cameras and corresponding algorithms for head tracking. Three studies use software libraries for face tracking, i.e., Lohan et al. (2011) use FaceAPI^10^, and Pereira et al. (2019), Pereira et al. (2020) GazeSense^11^. Hosada et al. (2004) do not mention their head tracking solution. These four publications do not provide enough information to assign them to a specific sub-category.

Individual Template-based. Template-based approaches use representative images of the head to calculate changes in position by matching the template with the current image of the human’s face recorded by cameras (Cazzato et al., 2020). Template-based methods are used by four publications. Nagai et al. (2003) and Nagai (2005) do not describe their algorithm in detail. Scassellati (2002) uses the Ratio Templates algorithm by Sinha (1996), which recognizes faces based on brightness values of the image and then compares them with the template. Da Silva and Romero (2012) use the Adaptive Appearance Model, an extension in which template images are taken from different perspectives (Morency et al., 2003).

Local Feature-based. By detecting colors, edges, and textures within the image, these approaches are based on the identification of specific, local features of the head, such as eyes, nose, or other landmarks. A generic model of the head, its proportions and facial features is then employed to detect the head orientation and position. A well-known and frequently used feature-based algorithm is that of Viola and Jones (2004). It is based on rectangular patterns, so-called Haar-like features, which are simple patterns that calculate the difference in intensity between neighboring rectangular regions in an image. These features are then used in a series of machine learning classifiers for recognizing faces in real time. Saran et al. (2018) uses the programming library Dlib, which implements this algorithm. Yücel et al. (2013) implement the algorithm themselves. In addition to this, there are other methods based on local facial features. Shon et al. (2005) start by determining the region of the face by localizing skin color. They generate a head model and store local image features such as edges and textures in it. Palinko et al. (2016) use the Constrained Local Model (CLM), calculating the head position in a similar manner by combining local features and a model of the head (Baltrusaitis et al., 2012). Similarly, Lombardi et al. (2022) estimate gaze-related cues from facial landmarks by extracting keypoints with OpenPose^12^ and classifying gaze direction (left/right) training a SVM. Kim et al. (2008) use OpenCV for face recognition and do not further specify the underlying algorithm. Building on this, they implement an enhanced approach for pose detection, which is analogous to their object recognition method and involves identifying and clustering local edges and facial features.

Vector Embedding-based. Similar to vector embedding-based object tracking, these methods employ deep learning models to generate high-dimensional vector representations of facial features, which are then used to determine head position in images. Four studies adopt a vector embedding-based approach. Deng et al. (2021) use a solution based on the CNN FaceNet architecture developed by Schroff et al. (2015). Hanifi et al. (2024) also implement a method based on the FaceNet architecture, combined with a neural network to create a gaze heatmap, enhancing gaze tracking capabilities. Samaniego et al. (2024) utilize the YOLO algorithm for face detection and a CNN VGG16 model for gaze estimation.Jin et al. (2022) propose an end-to-end deep-learning gaze-following model that predicts the attended target location in the image from the scene image together with the detected head/face region. They use a two-pathway CNN (scene image + cropped head), and during training add two branches: one predicts a depth map from the scene features and the other predicts 3D gaze orientation from the head features; the resulting depth/orientation features are fused back into the main network to improve the final gaze heatmap prediction.

Depth-based. These approaches use depth camera images and algorithmic models to analyze spatial information, on the basis of which head position and orientation is estimated in three-dimensional space. For instance, Fischer and Demiris (2016) use the random forest implementation for depth-based face position recognition by Fanelli et al. (2011).

### 3.3 Gaze–environment mapping

With the third functional dimension of gaze following systems, the human gaze and the environment are brought together in order to identify the gaze target and, in a final step, have the robot react accordingly. This mapping, i.e., the projection of the gaze onto objects, can be implemented in different ways. Five studies included in the present review do not specify how gaze–environment mapping is implemented, and can, thus, not be assigned to any of the technological subcategories (Lohan et al., 2011; Pereira et al., 2019; Pereira et al., 2020; Yoshikawa et al., 2006a; Yoshikawa et al., 2006b).

Gaze Vector-based. One way to identify the gaze target is to use a geometric strategy by elongating the gaze vector until it intersects with an object in the environment. This approach to gaze–environment mapping is used in eight studies (Xu et al., 2013; Willemse and Wykowska, 2019; Raković et al., 2024; Ravindra et al., 2009; Palinko et al., 2015; Shon et al., 2005; Scassellati, 2002; Samaniego et al., 2024). Lombardi et al. (2022) employ a coarse, vector-based strategy in which gaze direction (left/right) is used to select the relevant demonstrator hand, after which the object is detected in the hand. Two studies improve the gaze vector-based mapping by incorporating the robot’s ability to compute which objects are visible from the human’s viewpoint. This technique, called perspective taking, combines the gaze information with the spatial arrangement of the objects and their relative positions, allowing the robot to distinguish between visible and occluded objects (Marin-Urias et al., 2009; Fischer and Demiris, 2016). Deng et al. (2021) report a more reliable solution by adopting a multi-level vector-based mapping approach.

Saccade-based. Another approach involves analyzing saccades–rapid eye movements that shift gaze quickly from one point to another, typically between fixations–to estimate the visual target area. For example, vertical and horizontal displacement or velocity of saccadic eye movements can indicate gaze direction and, thus, the object of focus. Palinko et al. (2016) follow this approach, identifying saccades using fixed velocity thresholds on the (vertical) angular velocity of the estimated gaze signal and using them to infer the object of focus.

Gaze Probability-based. There are approaches that go beyond mechanical interpretations of gaze direction by introducing principles of human perception or attention, and by referring to psychological concepts such as human intention. These approaches include higher-level contextual information to infer what a human is most probably focusing on. For instance, some approaches predict gaze targets using probability distributions that integrate various data sources like perceptual saliency maps and gaze heatmaps. For instance, Yücel et al. (2013) estimate a gaze vector by combining gaze direction with depth measurements probabilistically and aligning the outcome with a local saliency map. Domhof et al. (2015) use an eye tracker to estimate gaze direction, generating a saliency map based on gaze data and combining it with an environment-based saliency map to determine the most likely target objects. Saran et al. (2018) use a neural network model to generate two gaze heatmaps, capturing referential and mutual gaze. Their system combines these heatmaps with contextual knowledge about object locations. Hanifi et al. (2024) take a similar approach by combining a gaze heatmap with object detection results to predict the gaze targets. Further advancing this approach, Jin et al. (2022) introduce a complex CNN that integrates depth and orientation data to estimate the object or person of interest within a scene.

Learning-based. This category includes solutions that allow robots to learn sensorimotor coordination skills, i.e., mapping facial patterns and gaze direction to corresponding motor outputs to enable joint attention, utilizing both supervised and reinforcement learning (RL) techniques. In supervised learning, robots develop a mapping between human gaze direction and gaze targets using labeled data. The learning process involves iteratively refining the model by minimizing the difference between the predicted and desired outcomes, thereby improving the model accuracy.Three studies use this approach: Nagai et al. (2003) implement a simple feed-forward, fully connected three-layer neural network. Hosada et al. (2004) introduce a self-organizing map in combination with a 2-layer forward fully connected network to decrease training time. Nagai (2005) adopts a motion-based strategy, i.e., motion and edge features are extracted from facial data across several camera frames and used to train two separate neural networks to control robot movements. Reinforcement learning, on the other hand, enables robots to learn behavior autonomously through trial and error. Through a reward system, robots are trained to repeat actions that lead to positive results while avoiding negative ones, gradually optimizing their performance. A RL approach is adopted by three studies: Kim et al. (2008) use the critic reinforcement learning scheme outlined by Triesch et al. (2007). Da Silva and Romero (2012) implement three different reinforcement learning algorithms, and Mahzoon et al. (2019) introduce two algorithms designed to enhance the autonomous learning of contingent behavior.

## 4 Discussion

The present scoping review provides an overview of technological approaches to implement gaze following capabilities in robots. To this end, a systematic literature search was performed including four databases. On the basis of several inclusion and exclusion criteria 28 studies were selected for the review. A taxonomy was developed to systematize the existing solutions with respect to three basic functional dimensions of gaze following systems, i.e., environment tracking, gaze tracking, and gaze–environment mapping. The environment and gaze tracking solutions comprise approaches that are constrained with respect to the interaction setting, and those that are unconstrained.

There is no clear preference for either constrained or unconstrained solutions in the field of environment tracking, as both approaches are equally represented in existing research. While reliable in controlled settings, the drawback of constrained solutions is that they can only be used in static laboratory environments and are not adaptable to changing, open environments. To overcome this limitation, gaze following systems need to have more advanced spatial capacities. As presented in this review, this can be achieved using global saliency maps, which dynamically capture the visual field by identifying and highlighting salient features within the environment. Additionally, unconstrained object detection is possible through the identification of local features of objects within the environment. The third option for unconstrained object detection uses deep learning methods with vector embeddings.

Moving on to tracking the human’s gaze direction, it is striking that the majority of studies use the orientation of human’s head as a proxy for gaze direction. While head tracking may be computationally efficient and easy to implement, head tracking and eye tracking are not equivalent with respect to precision. Head position provides only a rough estimate of the human gaze. Palinko et al. (2016) point out that predicting the human gaze on the basis of head position is less accurate which, in turn, leads to robots being perceived as less smart and efficient. They offer several explanations on why there is a lack of eye tracking solutions in social robotics. One reason are the hardware requirements, as high-resolution cameras with a narrow field-of-view are needed to track the eye accurately. In addition, a high network bandwidth is important to allow for real-time calculations. Although eye tracking can present technical challenges, with advances in computer vision technologies, affordable hardware and the increasing availability of artificial intelligence and deep learning algorithms, the potential for precise and efficient eye tracking solutions is growing. This review shows that it is possible to track the human eye and even combine eye tracking with head tracking using computer vision. These systems work remotely, i.e., no sensors need to be attached to the user.

While unconstrained solutions are more versatile, constrained tracking systems, such as those using head-mounted eye tracking glasses or head motion capture systems that require visual markers, are justified in certain cases. For example, it is not always possible or practical for humans and robots to face each other head-on, a prerequisite for solutions that track gaze based on cameras and computer vision algorithms. Furthermore, eye trackers offer very accurate solutions and can be regarded as the gold standard in measuring eye gaze patterns (Kaduk et al., 2023). For this reason in particular, eye trackers are used commonly by studies investigating the effects of gaze following behavior on interactional outcomes.

An important aspect of gaze following systems is the mapping of the gaze to the environment, which can be achieved through various approaches. It is striking that many studies rely on a relatively simple vector-based approach, which involves elongating the gaze trajectory until it meets a point of interest within the environment. While this method is computationally efficient, it does not account for dynamic or probabilistic elements of human gaze behavior. More advanced systems enable robots to learn the connection between gaze and the environment autonomously or use probability-based methods to determine the likelihood of gaze targets using deep learning. Given the rapid development of deep learning technologies, these approaches are particularly promising and will further improve the accuracy and efficiency of gaze–environment mapping in the future.

In summary, this review shows that gaze following systems are a highly relevant area of research for HRI. It is conspicuous, however, that this review includes only 29 studies, which indicates that further research activity is indicated. The limited attention to the topic may be due to the technical complexity of these systems, which is reflected in the wide range of implementations that emerge from the overview of the studies. Obviously, there is no single best solution for gaze and environment-tracking systems. The choice of method depends on the equipment available, the environment in which the system is used and the specific focus of the application.

### 4.1 Limitations

To our knowledge, this systematic review is the first to systematically synthesize and evaluate existing research on technical solutions that enable a robot to follow the gaze of a human interaction partner. Although compliant with the PRISMA guidelines for a scoping review, several limitations should be pointed out (Page et al., 2021). First, only studies that met the inclusion criteria were examined. Unpublished research and grey literature were excluded from the onset. Consequently, the range of available solutions may not have been presented in full. Furthermore, although the search was carried out by two people separately, the data extraction was carried out by the first author only. Extraction and synthesis of the results by two independent researchers can be assumed to further reduce the possibility of bias and make the results more robust. Finally, it should be noted that the systematic review was not preregistered. However, the detailed description of the review process allows complete transparency on the review and the results.

### 4.2 Directions for future research

As a result of the present review, a number of starting points for future research in the field of gaze following robots emerge. First, the fact that many studies present constrained solutions to gaze and object tracking indicates a potential for further research. Future research should focus on gaze following systems that are adaptable and capable of working efficiently in unstructured environments, leading to better collaboration between humans and robots. Advancing in this field is particularly relevant for the deployment of robots in dynamic environments, such as schools or retirement homes, where ensuring their effective operation is essential. Second, future research should adopt existing, state-of-the-art methods from computer vision research when developing gaze following systems, as these methods have been thoroughly tested and validated in the field of human gaze estimation. The survey published by Cazzato et al. (2020) provides a comprehensive synthesis of such approaches. An example are transformer-based architectures that have become widely used in computer vision since their introduction in 2017 (Vaswani et al., 2017). Given their ability to integrate contextual information and model complex scenes, exploring transformer-based (or hybrid) approaches may be a promising direction for more robust gaze-following in HRI. Third, all considered publications enable robots to track and respond to changes in people’s gaze in real time. One limitation that persists across the majority of the studies, though, is the lack of information regarding the response time (e.g., end-to-end latency from gaze shift detection to robot reaction onset) of the systems, a crucial factor in interaction (Launay et al., 2013). Consequently, the current literature does not yet allow deriving a latency range or typical reaction times for gaze-following responses in social HRI settings. Nevertheless, a small number of studies do provide reaction-time estimates (e.g., Xu et al. (2013): 657 ms; Willemse and Wykowska (2019): 127 ms; Raković et al. (2024): 1.12–1.36 s). In the future, studies should provide information on how quickly the robot is able to react to the human gaze, i.e., what delay time can be expected. Finally, a point of criticism across studies is the oftentimes insufficiently described technical implementation. Several publications report their head tracking approaches rather superficially and which did not allow for these studies to be categorized into the taxonomy. Examples are studies that only state that a programming library was used without further describing its configuration, i.e., underlying algorithm. In the future, standardized technical reporting systems should be established to enable subsequent studies to effectively build on solutions that have already been published.

## 5 Conclusion

Joint attention is an important aspect in human–human interaction. With the right technology, robots can be equipped with the ability to mimic this human capacity by following the human gaze towards objects within a shared environment and reacting appropriately. This capability is crucial for creating natural and effective interactions between humans and robots. The aim of this scoping review was, thus, to present different technical methods enabling robots to track and follow human gaze in interaction scenarios. To lay a foundation for future advances in this promising field of HRI, available literature reporting technical solutions was screened and selected based on predefined inclusion and exclusion criteria. 29 studies met the inclusion criteria and passed the quality assessment.

The synthesis of the studies revealed significant methodological heterogeneity, rendering a direct comparison of existing approaches difficult. To classify the studies systematically, a taxonomy was developed, that allowed for the categorization of the studies based on their technical contribution to three dimensions of gaze following systems, namely, the environment tracking, the gaze tracking, and the gaze–environment mapping. We hope that this taxonomy serves as a foundational framework for future endeavors in the field of gaze following robots.

In summary, this overview shows how far research has already come in recent years and how much potential there is for further improvements as hardware components and approaches to computer vision continue to develop. Given that empirical studies show that the gaze following behavior of robots is crucial for the quality of HRI and for the perception of the robot as a social entity (Pereira et al., 2019; Pereira et al., 2020; Willemse and Wykowska, 2019), it is to be expected that the field will receive more attention in the future.

## Data Availability

The original contributions presented in the study are included in the article/supplementary material, further inquiries can be directed to the corresponding author.

## Footnotes

1
https://www.scopus.com

2
https://www.ieeexplore.ieee.org

3
https://www.webofscience.com

4
https://www.pubmed.ncbi.nlm.nih.gov

5
https://www.zotero.org

6
http://www.artoolkitx.org

7
https://opencv.org

8
https://www.eyecomtec.com/3132-faceLAB

9
http://dlib.net

10
https://github.com/vladmandic/face-api

11
https://eyeware.tech

12
https://github.com/CMU-Perceptual-Computing-Lab/openpose

## References

[B1] Admoni H. Scassellati B. (2017). Social eye gaze in human-robot interaction: a review. J. Hum.-Robot Interact. 6, 25–63. 10.5898/JHRI.6.1.Admoni

[B2] Albawi S. Mohammed T. A. Al-Zawi S. (2017). “Understanding of a convolutional neural network,” in 2017 international conference on engineering and technology (ICET), 1–6. 10.1109/ICEngTechnol.2017.8308186

[B3] Baltrusaitis T. Robinson P. Morency L. (2012). “3D constrained local model for rigid and non-rigid facial tracking,” in *2012 IEEE conference on computer vision and pattern recognition* (providence, RI: IEEE), 2610–2617. 10.1109/CVPR.2012.6247980

[B4] Bruce N. D. B. Tsotsos J. K. (2009). Saliency, attention, and visual search: an information theoretic approach. J. Vis. 9, 5. 10.1167/9.3.5 19757944

[B5] Cazzato D. Leo M. Distante C. Voos H. (2020). When I look into your eyes: a survey on computer vision contributions for human gaze estimation and tracking. Sensors Basel, Switz. 20, 3739. 10.3390/s20133739 32635375 PMC7374327

[B6] Ceola F. Maiettini E. Pasquale G. Rosasco L. Natale L. (2021). “Fast object segmentation learning with kernel-based methods for robotics,” in 2021 IEEE international conference on robotics and automation (ICRA), 13581–13588. 10.1109/ICRA48506.2021.9561758

[B7] Chevalier P. Kompatsiari K. Ciardo F. Wykowska A. (2020). Examining joint attention with the use of humanoid robots-A new approach to study fundamental mechanisms of social cognition. Psychonomic Bull. and Rev. 27, 217–236. 10.3758/s13423-019-01689-4 31848909 PMC7093354

[B8] Da Silva R. Romero R. (2012). Modelling shared attention through relational reinforcement learning. J. Intelligent Robotic Syst. Theory Appl. 66, 167–182. 10.1007/s10846-011-9624-y

[B9] Deng F. Zhou Y. Song S. Jiang Z. Chen L. Su J. (2021). Say what you are looking at: an attention-based interactive system for autistic children. Appl. Sci. Switz. 11, 7426. 10.3390/app11167426

[B10] Domhof J. Chandarr A. Rudinac M. Jonker P. (2015). “Multimodal joint visual attention model for natural human-robot interaction in domestic environments,” in 2015 IEEE/RSJ international conference on intelligent robots and systems (IROS), 2406–2412. 10.1109/IROS.2015.7353703

[B11] Emery N. (2000). The eyes have it: the neuroethology, function and evolution of social gaze. Neurosci. and Biobehav. Rev. 24, 581–604. 10.1016/S0149-7634(00)00025-7 10940436

[B12] Fanelli G. Weise T. Gall J. Van Gool L. (2011). “Real time head pose etimation from consumer depth cameras,” in Pattern recognition. Editors Mester R. Felsberg M. (Berlin, Heidelberg: Springer Berlin Heidelberg), 101–110.

[B13] Fischer T. Demiris Y. (2016). “Markerless perspective taking for humanoid robots in unconstrained environments,” in 2016 IEEE international conference on robotics and automation (ICRA), 3309–3316. 10.1109/ICRA.2016.7487504

[B14] Grigorescu S. M. Macesanu G. (2017). “Human–robot interaction through robust gaze following,” in Information technology and computational physics. Editors Kulczycki P. Kóczy L. T. Mesiar R. Kacprzyk J. (Cham: Springer International Publishing), 165–178.

[B15] Hanifi S. Maiettini E. Lombardi M. Natale L. (2024). A pipeline for estimating human attention toward objects with on-board cameras on the iCub humanoid robot. Front. Robotics AI 11, 1346714. 10.3389/frobt.2024.1346714 39483489 PMC11524796

[B16] Hosada K. Sumioka H. Morita A. Asada M. (2004). Acquisition of human-robot joint attention through real-time natural interaction. 2004 IEEE/RSJ Int. Conf. Intelligent Robots Syst. (IROS) 3, 2867–2872. 10.1109/IROS.2004.1389844

[B17] Itti L. Koch C. Niebur E. (1998). A model of saliency-based visual attention for rapid scene analysis. IEEE Trans. Pattern Analysis Mach. Intell. 20, 1254–1259. 10.1109/34.730558

[B18] Jin T. Yu Q. Zhu S. Lin Z. Ren J. Zhou Y. (2022). Depth-aware gaze-following *via* auxiliary networks for robotics. Eng. Appl. Artif. Intell. 113, 104924. 10.1016/j.engappai.2022.104924

[B19] Jording M. Hartz A. Bente G. Schulte-Rüther M. Vogeley K. (2018). The “social gaze space”: a taxonomy for gaze-based communication in triadic interactions. Front. Psychol. 9, 226. 10.3389/fpsyg.2018.00226 29535666 PMC5834481

[B20] Judd T. Ehinger K. Durand F. Torralba A. (2009). “Learning to predict where humans look,” in 2009 IEEE 12th international conference on computer vision (Kyoto: IEEE), 2106–2113. 10.1109/ICCV.2009.5459462

[B21] Kaduk T. Goeke C. Finger H. König P. (2023). Webcam eye tracking close to laboratory standards: comparing a new webcam-based system and the eyelink 1000. Behav. Res. Methods 56, 5002–5022. 10.3758/s13428-023-02237-8 37821751 PMC11289017

[B22] Kim H. Jasso H. Deak G. Triesch J. (2008). A robotic model of the development of gaze following (7th IEEE International Conference on Development and Learning), 238–243. 10.1109/DEVLRN.2008.4640836

[B23] Launay J. Dean R. T. Bailes F. (2013). Synchronization can influence trust following virtual interaction. Exp. Psychol. 60, 53–63. 10.1027/1618-3169/a000173 22935329

[B24] Lohan K. S. Pitsch K. Rohlfing K. J. Fischer K. Saunders J. Lehmann H. (2011). “Contingency allows the robot to spot the tutor and to learn from interaction,” in 2011 IEEE international conference on development and learning (ICDL) (Frankfurt am Main: IEEE), 1–8. 10.1109/DEVLRN.2011.6037341

[B25] Lombardi M. Maiettini E. Tikhanoff V. Natale L. (2022). “Icub knows where you look: exploiting social cues for interactive object detection learning,” in 2022 IEEE-RAS 21st international conference on humanoid robots (Humanoids), 480–487. 10.1109/Humanoids53995.2022.10000163

[B26] Mahzoon H. Yoshikawa Y. Ishiguro H. (2019). Ostensive-cue sensitive learning and exclusive evaluation of policies: a solution for measuring contingency of experiences for social developmental robot. Front. Robot. AI 6, 2. 10.3389/frobt.2019.00002 33501019 PMC7806015

[B27] Maiettini E. Pasquale G. Rosasco L. Natale L. (2020). On-line object detection: a robotics challenge. Aut. Robots 44, 739–757. 10.1007/s10514-019-09894-9

[B28] Marin-Urias L. F. Sisbot E. A. Pandey A. K. Tadakuma R. Alami R. (2009). “Towards shared attention through geometric reasoning for human robot interaction,” in 2009 9th IEEE-RAS international conference on humanoid robots, 331–336. 10.1109/ICHR.2009.5379555

[B29] Matsumoto Y. Zelinsky A. (1999). “Real-time stereo face tracking system for visual human interfaces. In *Proceedings International Workshop on Recognition, Analysis, and Tracking of Faces and Gestures in Real-Time Systems* ,” in *Conjunction with ICCV’99 (Cat. No.PR00378)* (Corfu, Greece: IEEE comput. Soc), 77–82. 10.1109/RATFG.1999.799227

[B30] Morency L.-P. Sundberg P. Darrell T. (2003). “Pose estimation using 3D view-based eigenspaces,” in *2003 IEEE international SOI conference. Proceedings (Cat. No.03CH37443)* (Nice, France: IEEE comput. Soc), 45–52. 10.1109/AMFG.2003.1240823

[B31] Nagai Y. (2005). “The role of motion information in learning human-robot joint attention,” in Proceedings of the 2005 IEEE international conference on robotics and automation, 2069–2074. 10.1109/ROBOT.2005.1570418

[B32] Nagai Y. Hosoda K. Asada M. (2003). Joint attention emerges through bootstrap learning. Proc. 2003 IEEE/RSJ Int. Conf. Intelligent Robots Syst. (IROS 2003) (Cat. No.03CH37453) 1, 168–173. 10.1109/IROS.2003.1250623

[B33] Page M. J. McKenzie J. E. Bossuyt P. M. Boutron I. Hoffmann T. C. Mulrow C. D. (2021). The PRISMA 2020 statement: an updated guideline for reporting systematic reviews. BMJ 372, n71. 10.1136/bmj.n71 33782057 PMC8005924

[B34] Palinko O. Rea F. Sandini G. Sciutti A. (2015). “Eye gaze tracking for a humanoid robot,” in 2015 IEEE-RAS 15th international conference on humanoid robots (Humanoids), 318–324. 10.1109/HUMANOIDS.2015.7363561

[B35] Palinko O. Rea F. Sandini G. Sciutti A. (2016). Robot reading human gaze: why eye tracking is better than head tracking for human-robot collaboration. IEEE/RSJ International Conference on Intelligent Robots and Systems IROS, 5048–5054. 10.1109/IROS.2016.7759741

[B36] Pereira A. Oertel C. Fermoselle L. Mendelson J. Gustafson J. (2019). Responsive joint attention in human-robot interaction. IEEE/RSJ International Conference on Intelligent Robots and Systems IROS, 1080–1087. 10.1109/IROS40897.2019.8968130

[B37] Pereira A. Oertel C. Fermoselle L. Mendelson J. Gustafson J. (2020). “Effects of different interaction contexts when evaluating gaze models in hri,” in Proceedings of the 2020 ACM/IEEE international conference on human-robot interaction (New York, NY, USA: Association for Computing Machinery), 131–139. 10.1145/3319502.3374810

[B38] Raković M. Ferreira Duarte N. Marques J. Billard A. Santos-Victor J. (2024). The gaze dialogue model: nonverbal communication in hhi and hri. IEEE Trans. Cybern. 54, 2026–2039. 10.1109/TCYB.2022.3222077 36446005

[B39] Ravindra P. De Silva S. Tadano K. Lambacher S. G. S. H. Higashi M. (2009). “Unsupervised approach to acquire robot joint attention,” in 2009 4th international conference on autonomous robots and agents, 601–606. 10.1109/ICARA.2000.4803926

[B40] Redmon J. Divvala S. K. Girshick R. B. Farhadi A. (2015). “You only look once: unified, real-time object detection,” in 2016 IEEE conference on computer vision and pattern recognition (CVPR), 779–788.

[B41] Samaniego M. Atxa E. Rodriguez I. Lazkano E. (2024). “Pepper says: “i spy with my little eye”,” in 2024 IEEE-RAS 23rd international conference on humanoid robots (Humanoids), 53–59. 10.1109/Humanoids58906.2024.10769796

[B42] Saran A. Majumdar S. Short E. S. Thomaz A. Niekum S. (2018). Human gaze following for human-robot interaction. IEEE/RSJ International Conference on Intelligent Robots and Systems IROS, 8615–8621. 10.1109/IROS.2018.8593580

[B43] Scassellati B. (2002). Theory of mind for a humanoid robot. Aut. Robots 12, 13–24. 10.1023/a:1013298507114

[B44] Scassellati B. (2003). Investigating models of social development using a humanoid robot. Proc. Int. Jt. Conf. Neural Netw. 4, 2704–2709. 10.1109/IJCNN.2003.1223995

[B45] Schroff F. Kalenichenko D. Philbin J. (2015). “Facenet: a unified embedding for face recognition and clustering,” in 2015 IEEE conference on computer vision and pattern recognition (CVPR), 815–823. 10.1109/CVPR.2015.7298682

[B46] Shon A. Grimes D. Baker C. Hoffman M. Zhou S. Rao R. (2005). “Probabilistic gaze imitation and saliency learning in a robotic head,” in Proceedings of the 2005 IEEE international conference on robotics and automation, 2865–2870. 10.1109/ROBOT.2005.1570548

[B47] Sinha P. (1996). Perceiving and recognizing three-dimensional forms. USA: Ph.D. thesis, Massachusetts Institute of Technology.

[B48] Su H. Qi W. Chen J. Yang C. Sandoval J. Laribi M. A. (2023). Recent advancements in multimodal human–robot interaction. Front. Neurorobotics 17, 1084000. 10.3389/fnbot.2023.1084000 37250671 PMC10210148

[B49] Tricco A. C. Lillie E. Zarin W. O’Brien K. K. Colquhoun H. Levac D. (2018). PRISMA extension for scoping reviews (PRISMA-ScR): checklist and explanation. Ann. Intern. Med. 169, 467–473. 10.7326/M18-0850 30178033

[B50] Triesch J. Jasso H. Deák G. O. (2007). Emergence of mirror neurons in a model of gaze following. Adapt. Behav. 15, 149–165. 10.1177/1059712307078654

[B51] Vaswani A. Shazeer N. Parmar N. Uszkoreit J. Jones L. Gomez A. N. (2017). Attention is all you need. Int. Conf. Neural Inf. Process. Syst., 6000–6010. 10.48550/arXiv.1706.03762

[B52] Viola P. Jones M. J. (2004). Robust real-time face detection. Int. J. Comput. Vis. 57, 137–154. 10.1023/B:VISI.0000013087.49260.fb

[B53] Willemse C. Wykowska A. (2019). In natural interaction with embodied robots, we prefer it when they follow our gaze: a gaze-contingent mobile eyetracking study. Philosophical Trans. R. Soc. B Biol. Sci. 374, 20180036. 10.1098/rstb.2018.0036 30852999 PMC6452241

[B54] Xu T. Zhang H. Yu C. (2013). Cooperative gazing behaviors in human multi-robot interaction. Interact. Stud. 14, 390–418. 10.1075/is.14.3.05xu

[B55] Xu H. Wang T. Chen Y. Shi T. (2023). “A practical, robust, accurate gaze-based intention inference method for everyday human-robot interaction,” in 2023 IEEE international conference on systems, man, and cybernetics (SMC), 2054–2061. 10.1109/SMC53992.2023.10394422

[B56] Yoshikawa Y. Shinozawa K. Ishiguro H. Hagita N. Miyamoto T. (2006a). “Impression conveyance with responsive robot gaze in a conversational situtation,” in Roman 2006 - the 15th IEEE international symposium on robot and human interactive communication, 457–462. 10.1109/ROMAN.2006.314370

[B57] Yoshikawa Y. Shinozawa K. Ishiguro H. Hagita N. Miyamoto T. (2006b). “Responsive robot gaze to interaction partner,” in Robotics: science and systems, 37–43. 10.15607/rss.2006.ii.037

[B58] Yücel Z. Salah A. Meriçli C. Mericļi T. Valenti R. Gevers T. (2013). Joint attention by gaze interpolation and saliency. IEEE Trans. Cybern. 43, 829–842. 10.1109/TSMCB.2012.2216979 23047879

[B59] Zhang R. Saran A. Liu B. Zhu Y. Guo S. Niekum S. (2020). “Human gaze assisted artificial intelligence: a review,” in Proceedings of the twenty-ninth international joint conference on artificial intelligence (Yokohama, Japan: International Joint Conferences on Artificial Intelligence Organization), 4951–4958. 10.24963/ijcai.2020/689 PMC7476326 32901189

